# Supplementary material for: Effective implementation of the Sport Education Model in physical education: A meta-analysis of participant and intervention characteristics
Source: PLoS One. 2025 Oct 16;20(10):e0331228. doi: 10.1371/journal.pone.0331228 (PMC12530615; doi:10.1371/journal.pone.0331228)
Supplement: S2 Appendix — (DOCX) [file pone.0331228.s002.docx]

| **S1 Table. Detailed search strategy** | |  |
| --- | --- | --- |
| **Search database** | **Keyword** | **Number** |
| **Web of Science** | ((TI=("Sport Education Model" OR "Sport Education" OR "Sport season")) AND TS=("student*" OR "physical education" or "PE")) AND AB=("performance" OR "outcome" OR "achievement" OR "effect*" OR "influence" OR "experiment" OR "academic achievement" OR "exercise capacity" OR "physical fitness" OR "physical quality" OR "knowledge" OR "skills" OR "motivation" OR "interest" OR "attitude" OR "mental health") | 182 |
| **Scopus** | TITLE ( "Sport Education Model" OR "Sport Education" OR "Sport season" ) AND TITLE-ABS-KEY ( "student*" OR "physical education" OR PE ) AND TITLE-ABS-KEY ( "performance" OR "outcome" OR "achievement" OR "effect*" OR "influence" OR "experiment" OR "academic achievement" OR "exercise capacity" OR "physical fitness" OR "physical quality" OR "knowledge" OR "skills" OR "motivation" OR "interest" OR "attitude" OR "mental health" ) | 346 |
| **PubMed** | (("Sport Education Model"[Title] OR "Sport Education"[Title] OR "Sport season"[Title]) AND ("student*"[Title/Abstract] OR "physical education"[Title/Abstract] OR "PE"[Title/Abstract])) AND ("performance"[Title/Abstract] OR "outcome"[Title/Abstract] OR "achievement"[Title/Abstract] OR "effect*"[Title/Abstract] OR "influence"[Title/Abstract] OR "experiment"[Title/Abstract] OR "academic achievement"[Title/Abstract] OR "exercise capacity"[Title/Abstract] OR "physical fitness"[Title/Abstract] OR "physical quality"[Title/Abstract] OR "knowledge"[Title/Abstract] OR "skills"[Title/Abstract] OR "motivation"[Title/Abstract] OR "interest"[Title/Abstract] OR "attitude"[Title/Abstract] OR "mental health"[Title/Abstract]) | 29 |
| **EBSCO host (CINAHL with Full Text)** | TI ("Sport Education Model" OR "Sport Education" OR "Sport season") AND SU ("student*" OR "physical education" OR "PE") AND AB ("performance" OR "outcome" OR "achievement" OR "effect*" OR "influence" OR "experiment" OR "academic achievement" OR "exercise capacity" OR "physical fitness" OR "physical quality" OR "knowledge" OR "skills" OR "motivation" OR "interest" OR "attitude" OR "mental health") | 24 |
| **EBSCO host (SPORTDiscus with Full Text)** | TI ("Sport Education Model" OR "Sport Education" OR "Sport season") AND SU ("student*" OR "physical education" or PE) AND AB ("performance" OR "outcome" OR "achievement" OR "effect*" OR "influence" OR "experiment" OR "academic achievement" OR "exercise capacity" OR "physical fitness" OR "physical quality" OR "knowledge" OR "skills" OR "motivation" OR "interest" OR "attitude" OR "mental health") | 188 |
