# Supplementary material for: Effective implementation of the Sport Education Model in physical education: A meta-analysis of participant and intervention characteristics
Source: PLoS One. 2025 Oct 16;20(10):e0331228. doi: 10.1371/journal.pone.0331228 (PMC12530615; doi:10.1371/journal.pone.0331228)
Supplement: S5 Appendix — (DOCX) [file pone.0331228.s005.docx]

**Supplementary information 1: Result of the overall implementation effect of the Sport Education Model in Physical Education teaching**

**Supplementary information 2: Result of effects of the Sport Education Model on student PE learning with different study design**

**Supplementary information 3: Result of the effects of the Sport Education Model on cognitive and non-cognitive dimensions in students**

Note: A: Cogonitive dimension; B: Non-cogonitive dimension

**Supplementary information 4: Result of effects of the Sport Education Model on student PE learning with different stages**

**Supplementary information 5: Result of effects of the Sport Education Model on student PE learning with different learning experience**

**Supplementary information 6: Result of effects of the Sport Education Model on student PE learning with different class size**

**Supplementary information 7: Result of effects of the Sport Education Model on student PE learning with different intervention frequency**

**Supplementary information 8: Result of effects of the Sport Education Model on student PE learning with different duration of the session**

**Supplementary information 9: Result of effects of the Sport Education Model on student PE learning with different experimental period**

**Supplementary information 10: Result of effects of implementation fidelity of the Sport Education Model on student physical education learning**
