# Supplementary material for: Effective implementation of the Sport Education Model in physical education: A meta-analysis of participant and intervention characteristics
Source: PLoS One. 2025 Oct 16;20(10):e0331228. doi: 10.1371/journal.pone.0331228 (PMC12530615; doi:10.1371/journal.pone.0331228)
Supplement: S1 Appendix — (PDF) [file pone.0331228.s001.pdf]

Cover Letter (with rebuttal letter)

Dear Editor-in-Chief,

I am pleased to submit our manuscript entitled "Effective Implementation of the Sport Education Model in Physical Education: A Meta-Analysis of Participant and Intervention Characteristics" for your consideration for publication in PLOS ONE. We believe that our work makes a timely contribution by systematically evaluating the effects of the Sport Education Model (SEM) on students' physical education outcomes and by identifying key participant and intervention characteristics that moderate its effectiveness.

In addition, in order to ensure the comprehensiveness of the review and the validity of the conclusions, we have taken advice from your journal and included non-English literature in the review (Details can be found in the Inclusion Criteria).

Our meta-analysis synthesizes findings from 15 studies with a total of 2,890 participants, revealing that SEM significantly enhances physical education learning outcomes. Notably, our subgroup analyses indicate that the model is particularly effective among secondary school students, in small class sizes, and when students have no prior SEM experience. Moreover, the results suggest that an optimal SEM intervention consists of two sessions per week (each lasting at least 60 minutes) over no more than 18 sessions. These insights not only enrich the theoretical understanding of SEM's impact but also provide practical recommendations for the sustainable development of school sports programs.

The PLOS ONE of educational practices is a growing concern, and our study addresses this by offering evidence-based strategies that support the effective and sustainable implementation of physical education programs. We believe that the findings of our study align well with the journal's focus on sustainable development across diverse fields.

We confirm that this manuscript represents original work that has not been published elsewhere and is not under simultaneous consideration by any other journal. All authors have approved the manuscript, and there are no conflicts of interest to declare.

Thank you for considering our manuscript for publication in PLOS ONE. We look forward to your feedback and are happy to provide any further information needed.

Sincerely,

Junlong Zhang, zhangjunlong2413@gmail.com
Kim Geok Soh, kims@upm.edu.my
University Putra Malaysia
