# Supplementary material for: Effective implementation of the Sport Education Model in physical education: A meta-analysis of participant and intervention characteristics
Source: PLoS One. 2025 Oct 16;20(10):e0331228. doi: 10.1371/journal.pone.0331228 (PMC12530615; doi:10.1371/journal.pone.0331228)
Supplement: Attachment — Submitted filename: Response to reviewers.docx [file pone.0331228.s007.docx]

**Reviewer/editor comments and revisions**

(Note: The text marked in different colors represents the reviewer/editor's comments, while the black font indicates our revisions and responses.)

We sincerely thank the editorial team and reviewers for their thoughtful and constructive feedback. Although we were granted sufficient time for revision, we devoted extensive effort to revising the manuscript promptly and thoroughly. All suggested modifications have been carefully addressed, and the manuscript has undergone two rounds of internal review to ensure accuracy and completeness.

**Journal Requirements:**

1. Please ensure that your manuscript meets PLOS ONE's style requirements, including those for file naming.

Response:

Thank you for the comment. We have carefully revised the manuscript to ensure full compliance with PLOS ONE’s formatting requirements. Specifically, all headings have been adjusted to use sentence case and appropriate font size, and all file names have been updated according to the journal’s naming guidelines. These changes are visible in the formatting of the headings throughout the manuscript. Additionally, we reformatted the figures following the FITT format; figures have been removed from the main text (only titles remain), and all figures have been uploaded as separate files. Figure titles are now placed immediately after the paragraph where each figure is first cited.

2–4 (Funding Statement):

2. We note that the grant information you provided in the ‘Funding Information’ and ‘Financial Disclosure’ sections do not match. When you resubmit, please ensure that you provide the correct grant numbers for the awards you received for your study in the ‘Funding Information’ section.

3. Thank you for stating in your Funding Statement: [This study was supported by A Project Supported by the Education Ministry's Youth Fund Project for Humanities and Social Sciences Research of China (Grant No. 24YJC890001).] Please provide an amended statement that declares all the funding or sources of support (whether external or internal to your organization) received during this study, as detailed online in our guide for authors. Please also include the statement “There was no additional external funding received for this study.” in your updated Funding Statement.

4. We note that you have provided funding information that is currently declared in your Funding Statement. However, funding information should not appear in the Acknowledgments section or other areas of your manuscript. Please remove any funding-related text from the manuscript and let us know how you would like to update your Funding Statement.

Response:

Thank you for your detailed comments regarding the funding information. We have carefully addressed each of the issues raised as follows:

We have corrected the inconsistency between the ‘Funding Information’ and the ‘Financial Disclosure’ sections. The grant number [Grant No. 24YJC890001] now appears consistently and correctly in the ‘Funding Information’ section.

We have revised the Funding Statement to declare all sources of support received during the study. The updated Funding Statement is as follows:

**Funding:**

**This study was supported by the Education Ministry's Youth Fund Project for Humanities and Social Sciences Research of China (Grant No. 24YJC890001). There was no additional external funding received for this study.**

In accordance with journal policy, **we have** **removed the funding information from the Acknowledgments section of the manuscript**. Funding is now declared exclusively in the Funding Statement section, as requested.

We have included the revised funding statement in the **cover letter**. Please update the online submission form on our behalf.

We hope these revisions meet your requirements and sincerely appreciate your guidance in helping us align with PLOS ONE’s submission standards.

5. Please include captions for your Supporting Information files at the end of your manuscript, and update any in-text citations to match accordingly. Please see our Supporting Information guidelines for more information.

Response:

Thank you for your comment. We have now added clear and complete captions for all Supporting Information files at the end of the manuscript, as per the journal’s guidelines. In addition, all in-text citations referring to Supporting Information have been updated to ensure consistency and correct referencing.

6. “Please review your reference list to ensure that it is complete and correct. If you have cited papers that have been retracted, please include the rationale for doing so in the manuscript text, or remove these references and replace them with relevant current references…”

Response:
Thank you for your comment. We have carefully reviewed the entire reference list to ensure accuracy and completeness. No retracted articles are currently cited. Minor formatting corrections have been made where necessary, and a few outdated references have been replaced with more recent and relevant sources (e.g. No. 19). These changes have been reflected in both the revised manuscript and the updated reference list.

**Additional Editor Comments:**

1. The manuscript presents a meta-analysis investigating the impact of the Sport Education Model (SEM) on students’ physical education learning outcomes, with a focus on participant and intervention characteristics. The topic is highly relevant, especially for educators and policymakers seeking evidence-based practices in physical education. The study is well-structured, follows PRISMA guidelines, and attempts to contribute to understanding moderating factors that affect SEM efficacy.

However, while the paper is ambitious and has notable strengths, several critical concerns related to methodological rigor, reporting transparency, analytical limitations, and writing quality must be addressed.

Response:

Dear editor, on behalf of my co-authors, I would like to sincerely thank you for your thoughtful and constructive comments regarding our manuscript. We are grateful that you found the topic of our meta-analysis relevant and timely, especially for educators and policymakers who rely on evidence-based insights in physical education. Your acknowledgment of the manuscript's structure, adherence to PRISMA guidelines, and its attempt to investigate moderating factors influencing the efficacy of the Sport Education Model (SEM) is deeply appreciated.

At the same time, we take your critical feedback regarding methodological rigor, reporting transparency, analytical limitations, and writing quality with utmost seriousness. In response to these concerns, we have undertaken a comprehensive revision of the manuscript. Below are our detailed point-by-point responses to the revisions you suggested.

2. Certainty of Evidence (GRADE Assessment) – Serious Concern
   “The GRADE evaluation rates the certainty of evidence for both cognitive and non-cognitive outcomes as very low. This severely limits the confidence in the findings. The authors should either: a) justify why their conclusions are still meaningful despite low evidence certainty, or b) temper their recommendations and claims of practical applicability.”

Response:

We appreciate the editor’s critical insight regarding the very low certainty of evidence as indicated by our GRADE assessment. In response, we have taken two important steps:

(1) Justification of Significance Despite Low Certainty:

While the GRADE assessment revealed low confidence in the estimates due to issues such as risk of bias and heterogeneity, the statistically significant and consistent effect sizes observed across both cognitive and non-cognitive domains suggest meaningful practical relevance. These findings provide valuable directional evidence for educators and policymakers, particularly in the context of limited high-quality randomized trials in this area.

(2) Tempering Practical Claims:

We have revised several sections of the manuscript, especially the Abstract, Discussion, and Conclusion, to soften the strength of our recommendations. Phrases implying definitive or causal conclusions have been moderated, and we have clearly acknowledged the limitations posed by the low GRADE ratings. Additionally, we now emphasize the exploratory nature of our meta-analysis and recommend further high-quality studies to strengthen future evidence.

**The revised parts in the manuscript:**

**Abstract (Page 3):**

**Original:** *“This study offers practical recommendations for SEM implementation and theoretical support for the high-quality development of school sports.”*

**Revised:** *“This study offers preliminary recommendations for SEM implementation and provides exploratory insights that may inform the high-quality development of school sports, while acknowledging the current limitations in evidence certainty.”*

**Limitations (Page 33)**

**Original:** *“...revealing the positive impact of SEM on enhancing students' physical education learning.”*

**Revised:** *“...revealing the potential positive impact of SEM on enhancing students' physical education learning, although these findings should be interpreted with caution due to the low certainty of evidence.”*

**Conclusion (page 35):**

**Original:** *“This study provides frontline physical education teachers with recommendations for selecting participant and intervention characteristics for SEM implementation and offers theoretical support for the high-quality development of school sports through SEM.”*

**Revised:** *“This study provides preliminary guidance for frontline physical education teachers regarding SEM implementation under different conditions. However, due to the very low certainty of evidence, these suggestions should be interpreted cautiously and require further validation through high-quality randomized trials.”*

3. High Heterogeneity (I² = 98%) and Need for Deeper Exploration
   “The I² value of 98% indicates extremely high heterogeneity, which threatens the validity of the overall pooled estimate. Although subgroup analyses are presented, the authors must explore and report sources of heterogeneity more deeply (e.g., study design quality, cultural context, variation in implementation fidelity).

Response:

We fully acknowledge the extremely high heterogeneity (I² = 98%) and agree that this presents a challenge to the validity of pooled estimates. In response to your suggestion, we have taken the following actions:

We expanded our **Methods**, **Results** and **Discussion** sections to include deeper exploration of potential sources of heterogeneity. Specifically, we added subgroup analyses based on study design and variation in implementation fidelity (Methods: Page 11; Results: Page17 and Page 21-24; Discussion: Page 32-33).

4. More visual representation of heterogeneity in the main manuscript or supplementary materials would strengthen transparency.

Response:

Thank you for your helpful suggestion. In response, we have included additional visual representations of heterogeneity in the supplementary material. Specifically, S3 File (“The detailed results of the meta-analysis of the overall and different moderating variables effects”) now contains forest plots of subgroup comparison to enhance the transparency and interpretability of heterogeneity across studies.

5. The subgroup analyses (e.g., by session frequency or class size) are informative but risk Type I error due to multiple comparisons and small subgroup sizes.

Response:

Thank you for pointing out this important concern. In response, we have revised both the **Results** and **Discussion sections** to explicitly acknowledge the increased risk of Type I error associated with multiple subgroup comparisons and small sample sizes within subgroups.

In the **Results section**, we added the following statement:“Nevertheless, due to the small number of studies within the intervention frequency and class size subgroups, there is an increased risk of Type I error, and thus these findings should be interpreted with appropriate caution.”**(Page 20)**

In the **Discussion section**, we further addressed this issue by noting:

“The extremely high heterogeneity (I² = 98%) suggests that differences in study design and implementation fidelity may have significantly influenced the pooled estimates. Although subgroup analyses were conducted, the exploratory nature of these analyses must be acknowledged—particularly given the elevated risk of Type I error. Future meta-analyses should aim to more precisely model these moderators.” **(page 32-33: Line 620-630)**

We believe these revisions strengthen the transparency and methodological caution of our interpretations and align with best practices in meta-analytic reporting.

6. The authors should indicate whether interaction tests were performed between subgroups and report corresponding p-values.

Response:

We sincerely appreciate this insightful suggestion. Conducting interaction tests between subgroups would indeed provide a more rigorous understanding of whether the observed subgroup differences are statistically meaningful. However, due to the limited number of studies within certain subgroups and the resulting low statistical power, we chose not to perform formal interaction tests in the current analysis to avoid overinterpretation of unstable estimates.

That said, we fully acknowledge the value of such analyses and agree that future meta-analyses with a larger pool of eligible studies would benefit from including formal interaction tests to better identify and quantify potential effect modifiers. We have now mentioned this as a direction for future research in the Discussion section.

7. The classification of outcomes into “cognitive” and “non-cognitive” needs clearer operational definitions. It is currently unclear, for instance, why “motor skills” are categorized under cognitive outcomes when they also depend on psychomotor learning.

Response:

We sincerely appreciate the reviewer’s thoughtful comment regarding the clarity of our outcome classification. We acknowledge that, to date, the academic community has not yet reached a universally agreed-upon distinction between “cognitive” and “non-cognitive” outcomes, particularly in the context of physical education research.

To address this concern, we have provided explicit operational definitions of both cognitive and non-cognitive outcomes under the “Eigenvalue coding and data extraction” subsection (page 10, highlighted in red in the revised manuscript). Our classification approach is consistent with that used in previous meta-analyses, particularly the work by Zhao et al. (2024), who categorized similar outcome variables along cognitive and non-cognitive lines within the context of physical education. This study has been cited as reference [28] in our manuscript:

Zhao, M., Lu, X., Zhang, Q., et al. (2024). Effects of exergames on student physical education learning in the context of the artificial intelligence era: a meta-analysis. *Scientific Reports*, 14(1), 7115. https://doi.org/10.1038/s41598-024-57357-8

We believe this clarification improves the transparency of our coding strategy and aligns our work with prior literature in this field.

8. While RoB-2 and ROBINS-I were used, the specific domains of bias (e.g., blinding, selection bias) are not discussed in sufficient depth.

Response:

Thank you for this insightful observation. We agree that providing a deeper discussion of the specific domains assessed by RoB-2 and ROBINS-I would strengthen the rigor and interpretability of our findings. In response, we have added two dedicated paragraphs **(Page 23-24: Line 417-434)** in the **Discussion section** to reflect on how common sources of bias-such as random sequence generation, lack of blinding, confounding, and missing data-may have influenced the effect estimates in the included studies.

This discussion elaborates on the differential risk profiles observed across randomized and non-randomized studies and highlights how these factors may explain variations in effect sizes. By including this analysis, we aim to improve the methodological transparency of our review and help readers better evaluate the confidence that can be placed in the pooled estimates.

The revised paragraph also references relevant literature (e.g., Flemyng et al., 2023; Sterne et al., 2016) to support the interpretation of domain-specific bias and its potential influence on systematic review findings.

9. Figure references are made (e.g., Fig 2, Fig 3), but visuals are not included in the provided material. Ensure these are present in the final submission.

Response:
Thank you for your attention to this issue. In accordance with the journal’s formatting requirements, we have removed all figures from the main manuscript and retained only the figure titles in the appropriate locations. All figures have been converted to high-resolution **TIFF format** and uploaded separately through the submission system as required. We have also ensured that each figure is clearly labeled and corresponds accurately to the in-text references in the revised manuscript.

10. The inclusion of non-English language articles is commendable, but more detail is needed about how translations were verified and whether this affected coding reliability.

Response:

Thank you for your thoughtful comment. We have added a clarifying statement in the Eligibility Criteria section of the Methods to address this point. **(Page 8)** Specifically, although our search strategy was conducted in English-language databases (e.g., Web of Science, Scopus, PubMed), we did not apply any language restrictions during screening. Studies with English titles and abstracts were considered regardless of the language of the full text.

For non-English full-text articles, preliminary interpretation was conducted using Google Translate by two reviewers (YG and JZ), and when necessary, verification was carried out by bilingual team members. Throughout the process, no significant difficulties were encountered in translating or interpreting eligible studies, and all extracted data were independently coded by two reviewers to ensure consistency. As such, no studies were excluded solely due to language barriers.

We believe this addition enhances the transparency and replicability of our study selection and coding procedures.

11. While overall readable, the manuscript contains several grammatical errors and awkward phrasings.The term “learning efficiency” is used without clear definition and appears non-standard. Consider replacing with “learning outcomes” or “educational gains.”

Response:
Thank you for your valuable feedback regarding language clarity and terminology. In response, we have thoroughly revised the manuscript to improve grammar, sentence structure, and overall readability. These edits have been implemented throughout the text and are marked using track changes.

Regarding the term “learning efficiency”, we agree that it is not a standard term within the physical education or educational research literature. Therefore, to ensure consistency and conceptual clarity, we have replaced all instances of “learning efficiency” with the more widely accepted term “learning outcomes” throughout the manuscript.

We appreciate the reviewer’s careful attention, which helped improve both the linguistic and conceptual quality of the paper.

12. Some appendices (e.g., Appendix 3 – description of 430 records) are critical for replication but should be summarized in the main text or tables for clarity.

Response:

Thank you for your suggestion. We agree with your comment and have added a summary row at the bottom of S1 File, which outlines the reasons for the inclusion and exclusion of the 430 full-text articles assessed for eligibility, to improve clarity and transparency.

The added summary line reads: "Summary of exclusions: Non-RCT or quasi-experimental studies (187); Not SEM intervention (62); Control group not standard/traditional teaching (59); Nonequivalent control groups (27); Conference papers (21); Reviews (21); Irrelevant populations (15); Book chapters (11); Full text not available (6); Data unavailable (4); Erratum (2); Final inclusion (15)."

We believe this addition helps readers better understand the study screening process at a glance.

13. The search was concluded in February 2025, and some included studies are listed as from 2024. Confirm the accuracy and peer-reviewed status of these articles.

Response:

Thank you for this important observation. We confirm that all studies included in the meta-analysis, including those listed as published in 2024, were formally published in peer-reviewed journals and were available in their final, citable form prior to the search cutoff date in February 2025.

The 2024 publication year refers to the official journal issue date, but these articles were already available online ahead of print (as early access or “online first” publications) and indexed in databases such as PubMed, Web of Science, and Scopus before our final search. We verified that each included study met the criteria of being peer-reviewed, published, and accessible in full text at the time of screening.

To further ensure transparency, we have rechecked all included studies and confirmed that no preprints or non–peer-reviewed sources were included.

14. The authors recommend 2 lessons/week for ≤18 sessions universally. However, contextual constraints (e.g., curriculum length, teacher capacity) should be acknowledged.

Response:

Thank you for this insightful comment. We agree that practical implementation of the recommended lesson frequency and duration may be influenced by contextual factors such as national curriculum structures, available instructional time, and teacher capacity.

In response, we have revised the **Practical Applications section** (Page33-34) to explicitly acknowledge these potential constraints. We now clarify that the recommendation of two lessons per week for no more than 18 sessions should be interpreted as a flexible, evidence-informed guideline rather than a rigid standard. The revised paragraph emphasizes the need for educators to adapt SEM implementation based on their specific institutional and instructional contexts.

This addition enhances the practical relevance of our findings and aligns the recommendations more closely with real-world educational settings.

Reviewer #1: The manuscript presents relevant findings on the effectiveness of the Sport Education Model. The methodology is generally appropriate, and the analysis is clear. Please ensure that all journal formatting and reference guidelines are carefully followed before final submission.

Response:

We sincerely thank you for your positive evaluation of our manuscript. We greatly appreciate your recognition of the relevance of our research results, the appropriateness of our methodology, and the clarity of our analysis.

Additionally, we have carefully reviewed and revised the manuscript again to ensure full compliance with all journal formatting and reference guidelines, including font styles, sentence capitalization, and file naming conventions. Your feedback has been very valuable in helping us improve the quality and presentation of our work. Thank you again.

Reviewer #2: I had the pleasure of participating in the review process of a manuscript entitled "Effective Implementation of the Sport Education Model in Physical Education: A Meta-Analysis of Participant and Intervention Characteristics."

The study investigates the facilitative effects of SEM on students' physical education learning and examines the Participant and Intervention Characteristics that modulate its impact.

The study provides novel research findings that significantly enhance the existing knowledge on the subject. A series of meticulously designed experiments were conducted, and the resulting data underwent rigorous statistical analyses, all executed to elevated technical standards. The author provides sufficient detail about the methods used in these analyses to ensure their comprehension and replication. The study's conclusions are obvious and based on the evidence. The author writes the document in typical academic English, demonstrating careful attention to word choice and order. It also follows the correct regulations for reporting (CONSORT, PRISMA...).

For these reasons, I believe that the manuscript is suitable for publication in its current form.

Response:

We sincerely appreciate your thoughtful and positive evaluation of our manuscript entitled "Effective Implementation of the Sport Education Model in Physical Education: A Meta-Analysis of Participant and Intervention Characteristics."

We are grateful for your recognition of the novel research findings, the rigorous experimental design, and the thorough statistical analyses conducted in this study.

Thank you also for acknowledging the clarity of our methodological descriptions and the adherence to established reporting guidelines such as CONSORT and PRISMA.

Your encouraging comments regarding the writing quality and overall suitability of the manuscript for publication mean a great deal to us.

We deeply appreciate your time and effort in reviewing our work and your valuable feedback.
